# Supplementary material for: Transgenerational Drought and Methyl Jasmonate Memory Interactively Shape Metabolome and Physiology in Clonal Grass
Source: Physiol Plant. 2025 Dec 30;178(1):e70720. doi: 10.1111/ppl.70720 (PMC12752454; doi:10.1111/ppl.70720)
Supplement: Supplementary file 1 — Figure S1: Correlation heatmap showing the relationship among chlorophyll fluorescence parameters measured during the stress phase (CFS1, CFS2), recovery phase (CFR1, CFR2), chlorophyll content measured during the stress phase (CCS1, CCS2), and recovery phase (CCR1, CCR2). Correlation coefficients are color‐coded from dark blue (low correlation) to dark red (high correlation), with values displayed within each cell. Figure S2: Boxplots showing the effects of interactions of (a) MeJA memory (M1) and drought memory (D1) on Hill evenness in positive mode and (b) current drought (D2) on Hill evenness negative mode in F. rubra . The X axis shows the levels of the interacting treatment, with 0 indicating absent, and 1 present treatment (drought or MeJa). Figure S3: Correlation heatmap showing the relationship among Richness, Shannon diversity, and Hill evenness. Correlation coefficients are color‐coded from dark blue (low correlation) to dark red (high correlation), with values displayed within each cell. Figure S4: Boxplots showing the effects of interactions M1, D1, D2, that is, MeJA memory, drought memory and current drought, respectively, in positive mode for (a) richness, (b) Shannon diversity, and (c, d) uniqueness in F. rubra . The X axis shows the levels of the interacting treatment, with 0 indicating absent and 1 present treatment (drought or MeJA). Figure S5: Boxplots show the distribution of metabolic uniqueness scores for each treatment group under negative ionization mode, representing the triple interaction of drought memory, current drought, and MeJA memory. In each code, a 1 in the first position indicates the presence of drought 1 (D1), a 1 in the second position indicates the presence of drought 2 (D2), and a 1 in the third position indicates the presence of the morphological modifier (M1). For example, 1_0_0 represents D1 only, 0_1_0 represents D2 only, and 1_1_1 represents a combination of D1, D2, and M1. The control group is 0_0_0, where no drought or [file PPL-178-e70720-s001.pdf]

Supplementary Information

Transgenerational drought and methyl jasmonate memory interactively shape metabolome and physiology in clonal grass

Tarun Bhatt[1,2*], Nikita Rathore[2], Jaroslav Semerád [3,4], Tomáš Cajthaml[3,4], Dinesh Thakur[2†], Zuzana Münzbergová[1,2†]

[1]Department of Botany, Faculty of Sciences, Charles University, Benátská 2, 12800, Prague, Czech Republic, 12800

[2]Institute of Botany, Czech Academy of Sciences, Zámek 1,25243, Průhonice, Czech Republic

[3]Institute of Microbiology of the Czech Academy of Sciences, Vídeňská 1083, 142 20 Prague, Czech Republic

[4]Institute for Environmental Studies, Faculty of Science, Charles University, Albertov 6, 12800, Prague 2, Czech Republic

[†]Senior authors

*Correspondence E-mail: bhattt@natur.cuni.cz

Figure S1: Correlation heatmap showing the relationship among chlorophyll fluorescence parameters measured during the stress phase (CFS1, CFS2), recovery phase (CFR1, CFR2), chlorophyll content measured during the stress phase (CCS1, CCS2), and recovery phase (CCR1, CCR2). Correlation coefficients are color-coded from dark blue (low correlation) to dark red (high correlation), with values displayed within each cell.

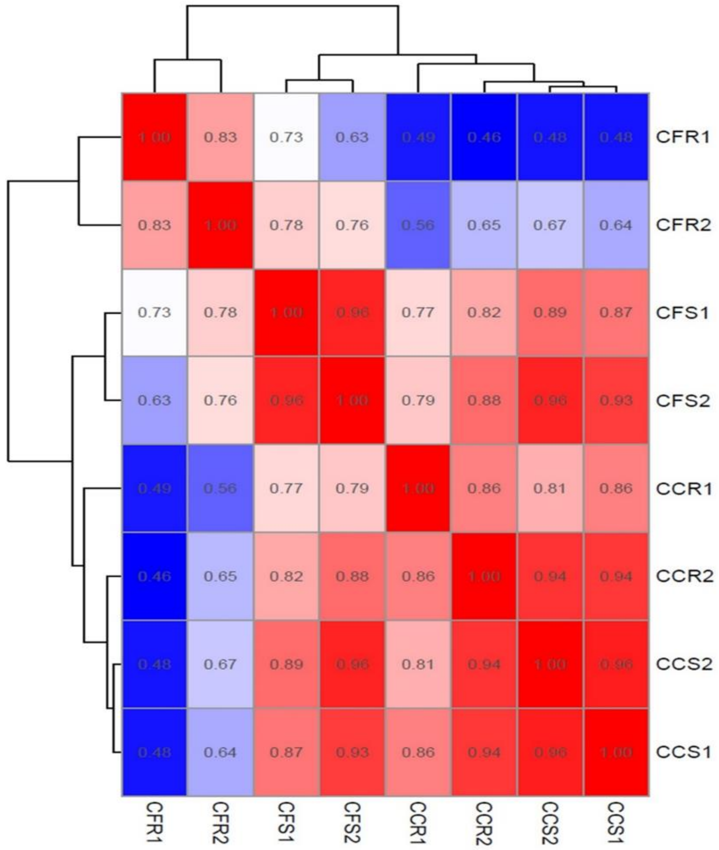

Figure S2: Boxplots showing the effects of interactions of (a) MeJA memory (M1) and drought memory (D1) on Hill Evenness in positive mode and (b) current drought (D2) on Hill Evenness negative mode in *F. rubra*. The X axis shows the levels of the interacting treatment, with 0 indicating absent and 1 present treatment (drought or MeJa).

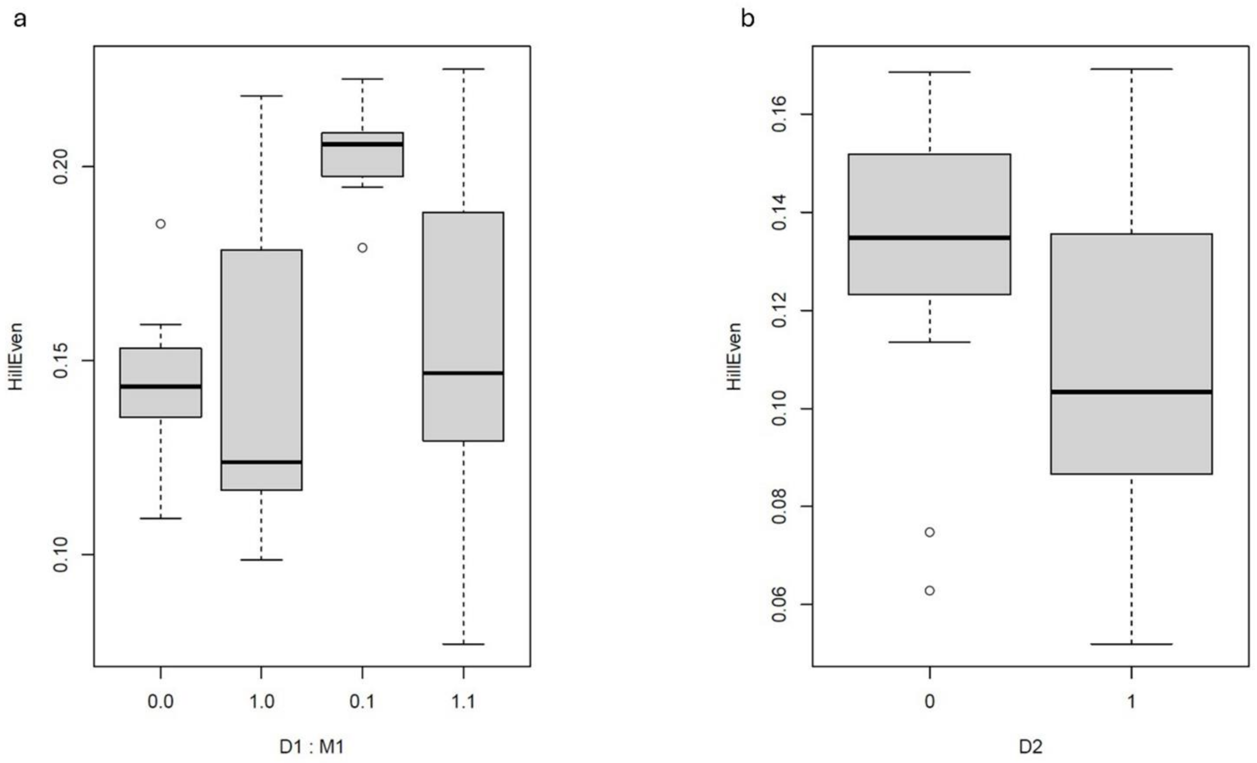

Figure S3: Correlation heatmap showing the relationship among Richness, Shannon diversity, and Hill Evenness. Correlation coefficients are color-coded from dark blue (low correlation) to dark red (high correlation), with values displayed within each cell.

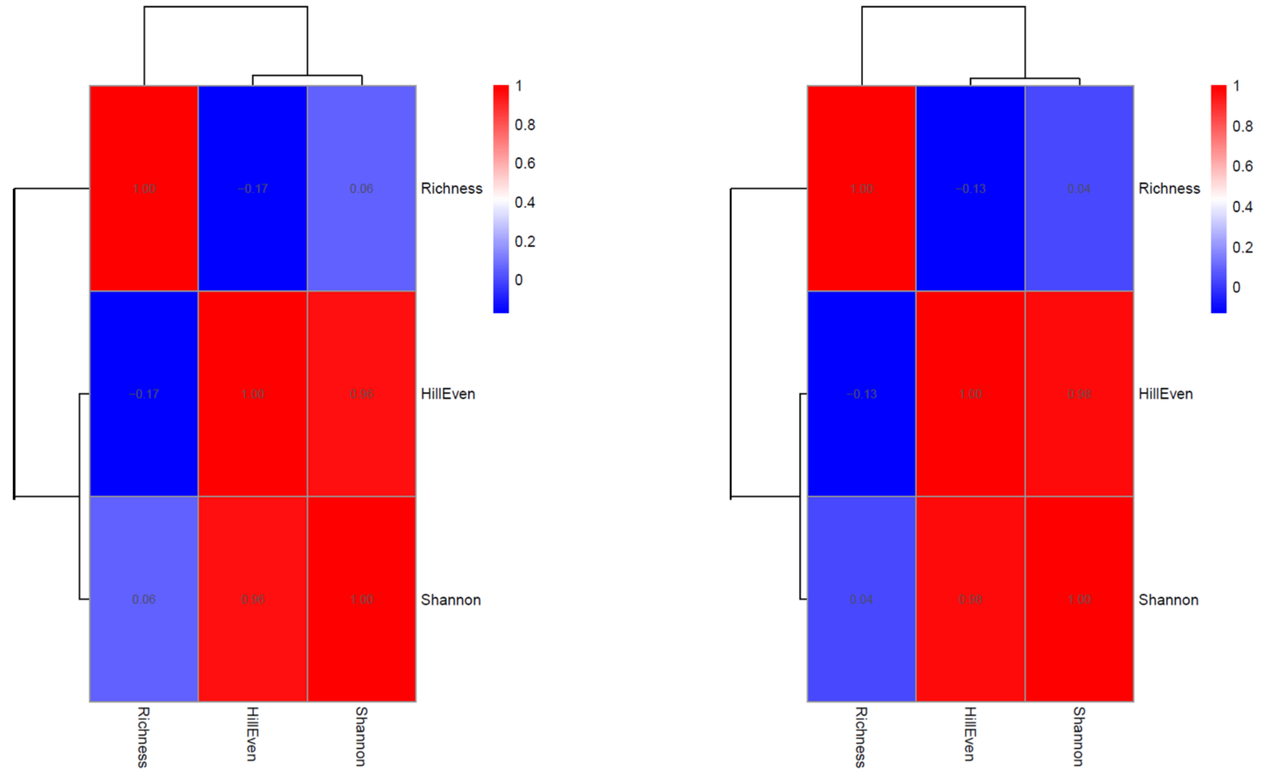

Figure S4: Boxplots showing the effects of interactions M1, D1, D2, i.e. MeJA memory, drought memory and current drought, respectively, in positive mode for (a) Richness, (b) Shannon diversity, and (c and d) Uniqueness in *F. rubra*. The X axis shows the levels of the interacting treatment, with 0 indicating absent and 1 present treatment (drought or MeJA).

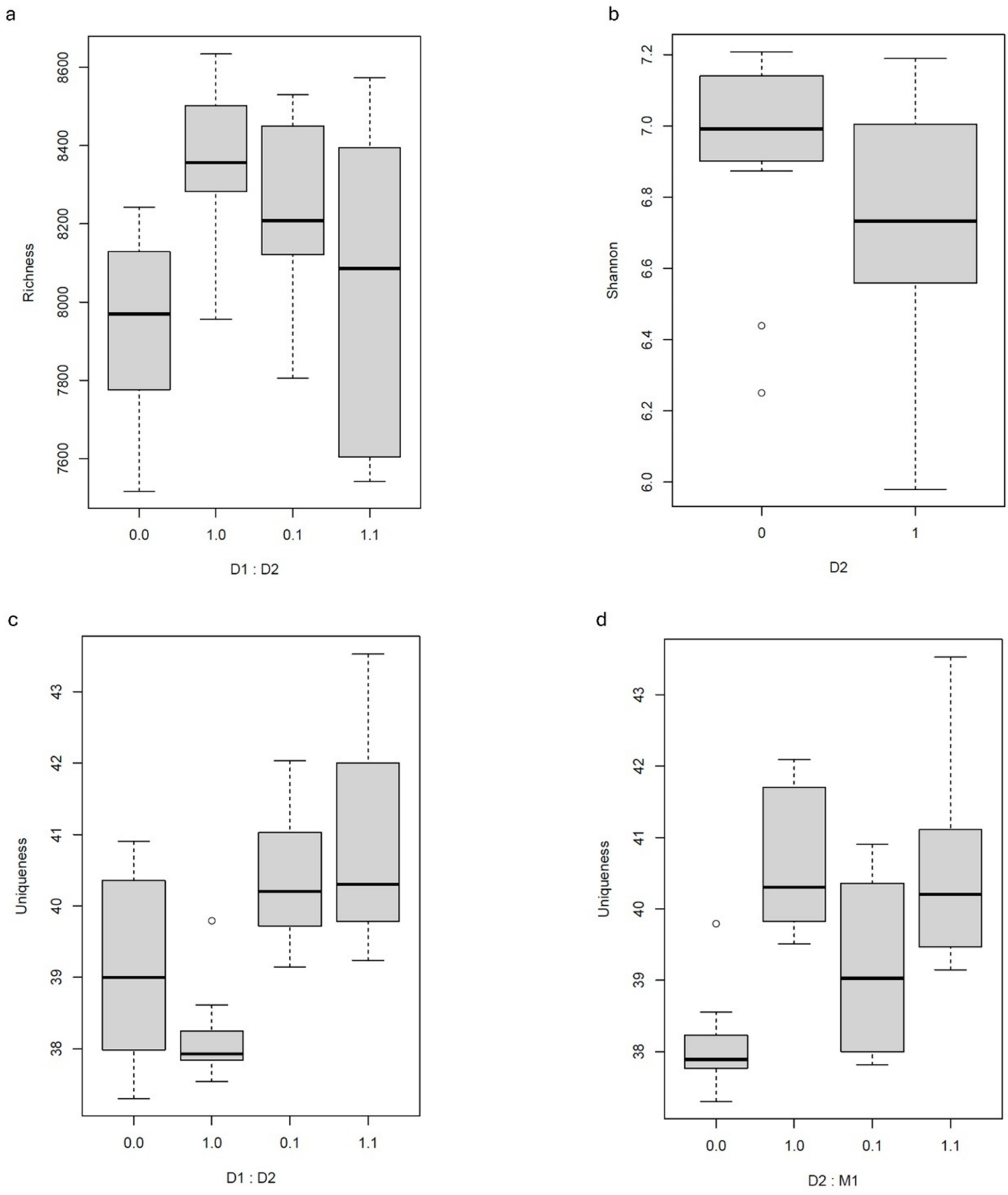

Figure S5: Boxplots show the distribution of metabolic uniqueness scores for each treatment group under negative ionization mode, representing the triple interaction of drought memory, current drought, and MeJA memory. In each code, a 1 in the first position indicates the presence of drought 1 (D1), a 1 in the second position indicates the presence of drought 2 (D2), and a 1 in the third position indicates the presence of the morphological modifier (M1). For example, 1_0_0 represents D1 only, 0_1_0 represents D2 only, and 1_1_1 represents a combination of D1, D2, and M1. The control group is 0_0_0, where no drought or modifier is applied.

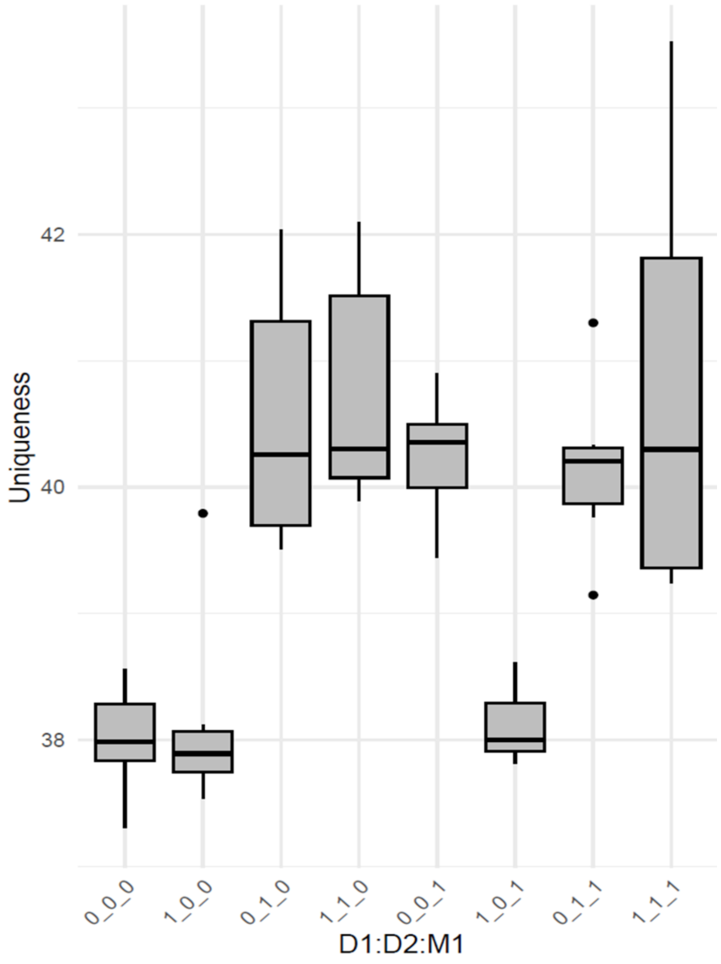

Figure S6: The RDA plots both ionization modes (**a**) positive and (**b**) negative, providing a clear view of the variation in metabolite profiles explained by M1, D1, D2, i.e., MeJA memory, drought memory and current drought, respectively.

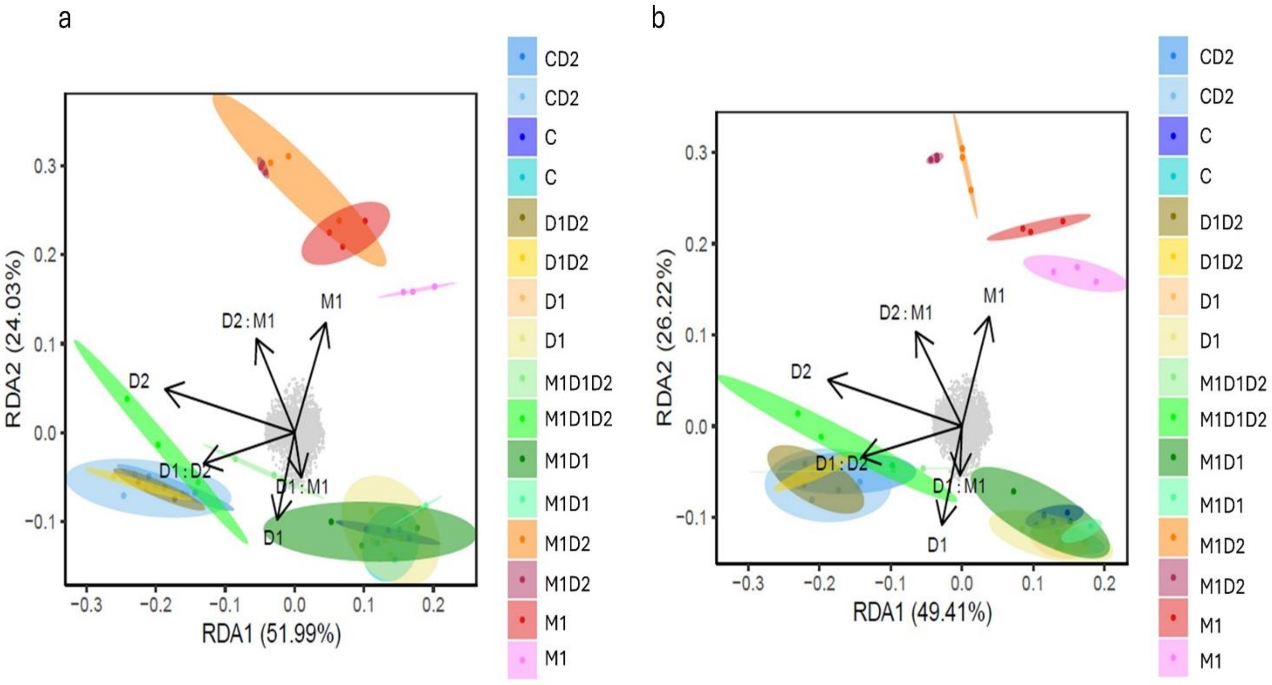

Figure S7: Bar plots representing the number of significant metabolites associated with drought memory (D1), MeJA memory (M1), current drought (D2), and their interactions (D1×D2, D1×M1, D2×M1) under positive ionization mode (a) and negative ionization mode (b). Each treatment or interaction is shown with two bars: shared metabolites (orange) indicate those common with other conditions, while unique metabolites (green) represent those specific to a given condition or interaction. The values above each bar indicate the exact number of metabolites in each category.

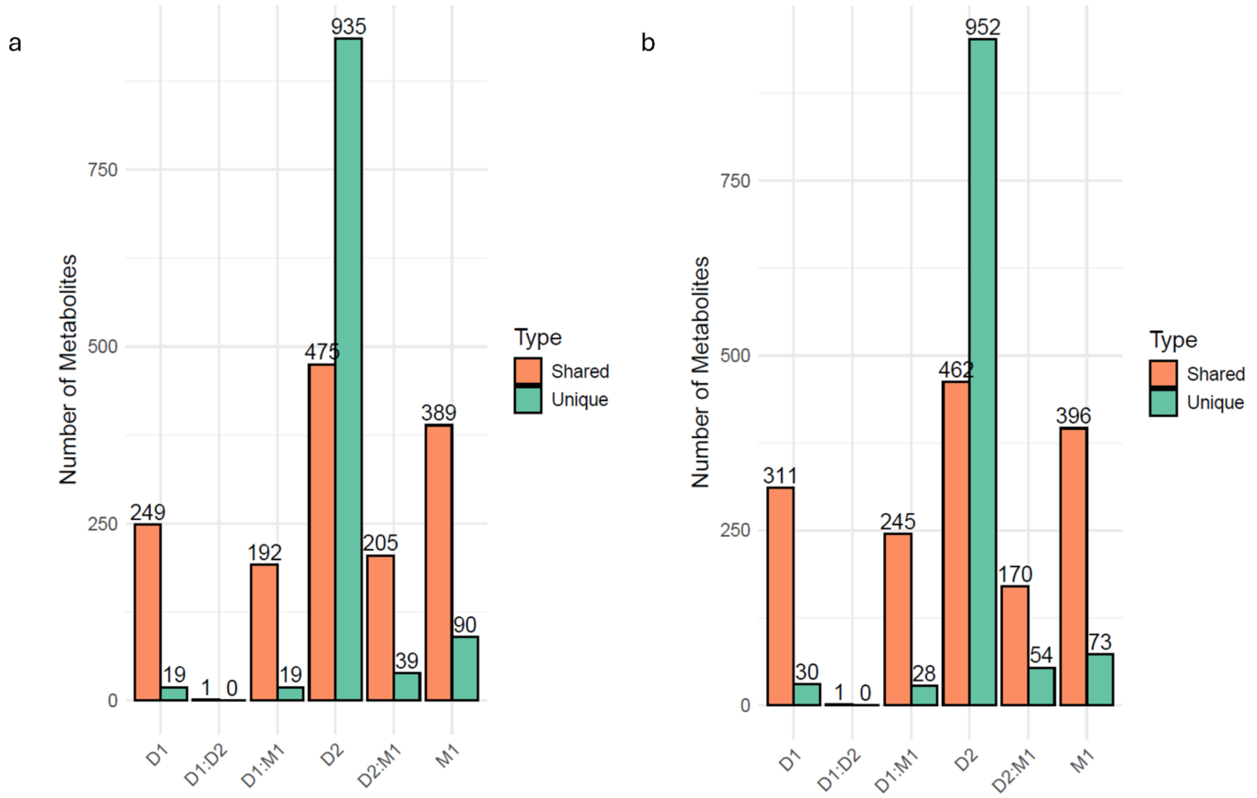

Figure S8: Venn diagram representing the number of significant metabolites associated with drought memory (D1), MeJA memory (M1), current drought (D2), and their interactions (D1×M1, D2×M1) under positive ionization mode (a) and negative ionization mode (b). We excluded D1×D2 from the Venn diagram to make it less complex, as it has only 1 metabolite.

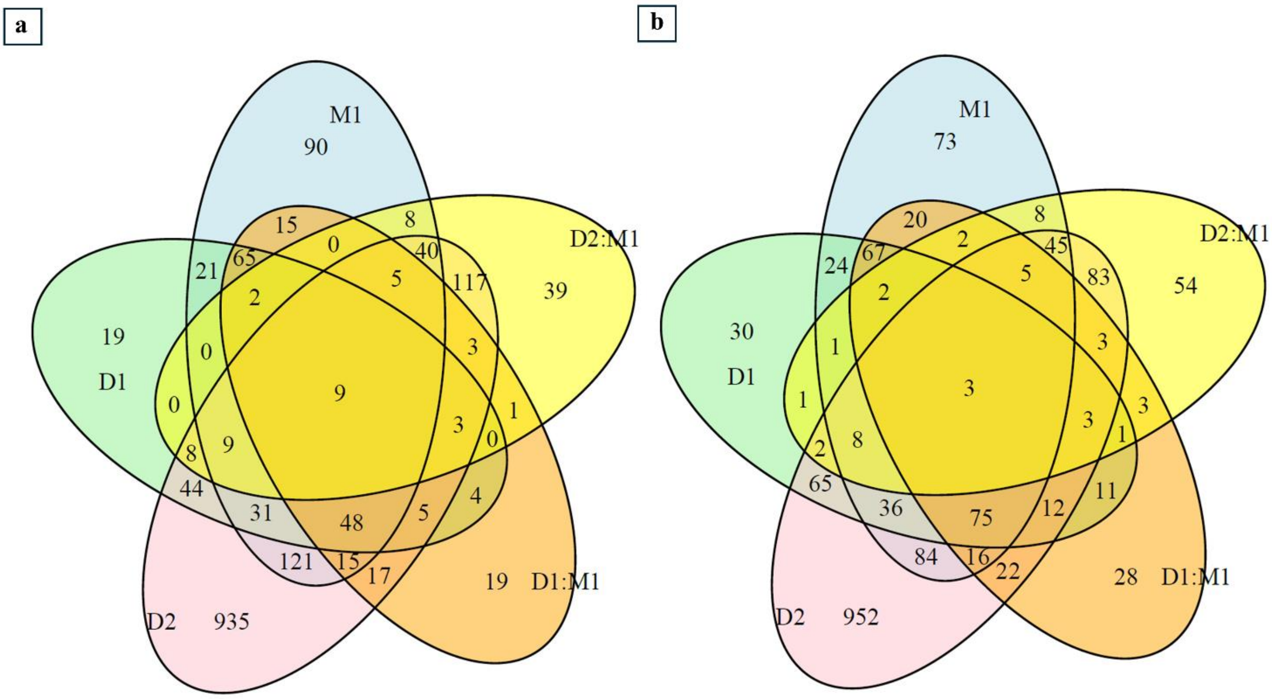

Figure S9: Heatmap displaying the scaled intensity values of differentially accumulated metabolites across various treatment groups in *F. rubra* (M1, D1, D2, i.e. MeJA memory, drought memory and current drought, respectively), others are the plants which got combination M1, D2 and D2 treatment, while missing code in combination indicates that plants where not treated by the given treatment. C indicates Control, i.e., no drought or MeJa treatment memory, and in well-watered conditions. Heatmap a to d shows cluster 1 to 4, respectively, for positive mode, while heatmap e and f shows cluster 1 and 2, respectively, for negative mode. The orange color in the plot represents a higher accumulation of a metabolite, while the blue color represents a lower accumulation.

a

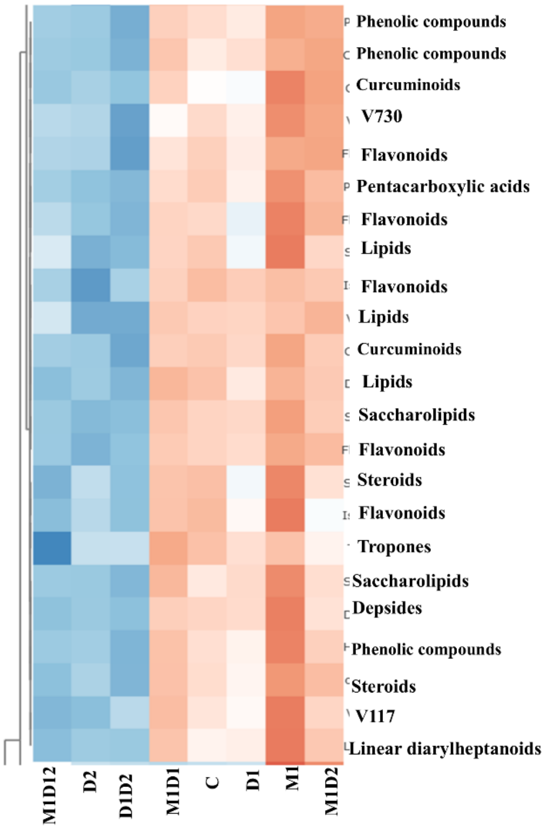

b

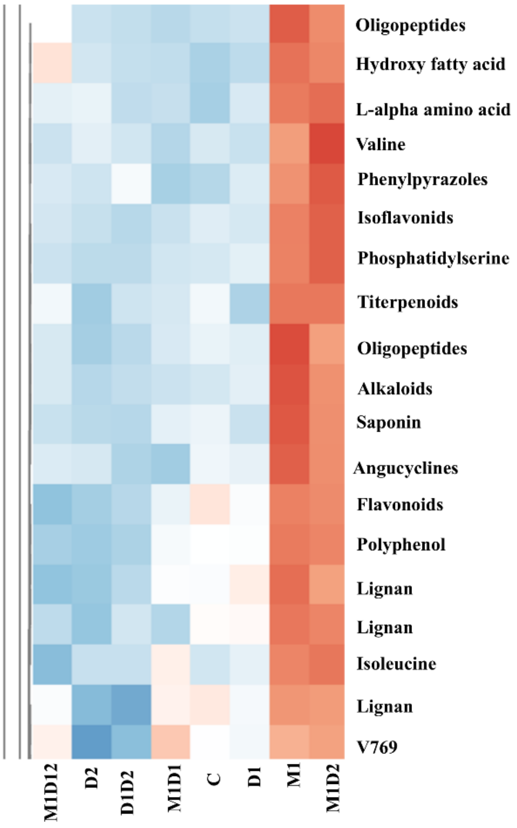

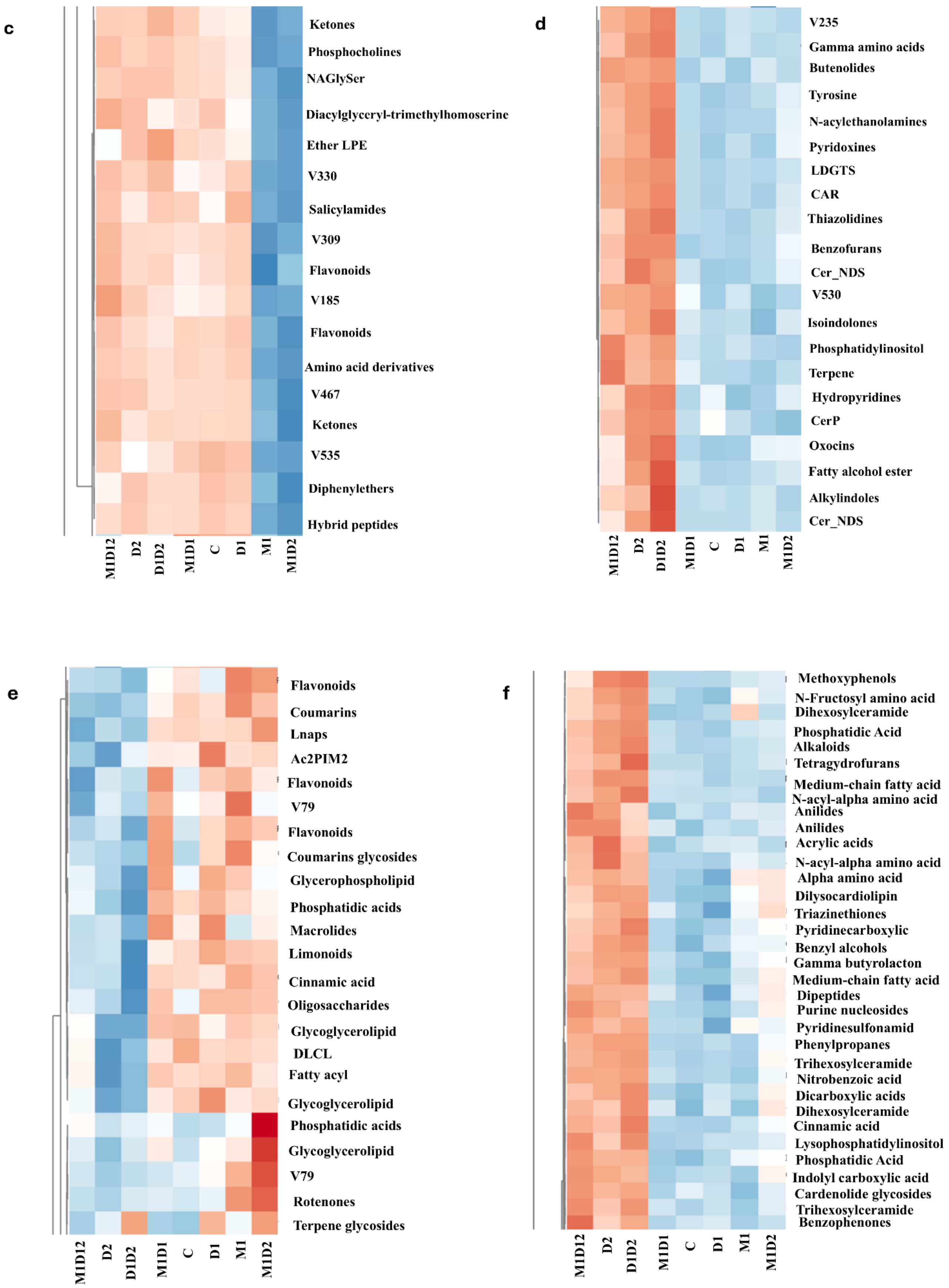
c
Ketones
Phosphocholines
NAGlySer
Diacylglyceryl-trimethylhomoserine
Ether LPE
V330
Salicylamides
V309
Flavonoids
V185
Flavonoids
Amino acid derivatives
V467
Ketones
V535
Diphenylethers
Hybrid peptides
M1D12
D2
D1D2
M1D1
C
D1
M1
M1D2
d
V235
Gamma amino acids
Butenolides
Tyrosine
N-acylethanolamines
Pyridoxines
LDGTS
CAR
Thiazolidines
Benzofurans
Cer_NDS
V530
Isoindolones
Phosphatidylinositol
Terpene
Hydropyridines
CerP
Oxocins
Fatty alcohol ester
Alkylindoles
Cer_NDS
M1D12
D2
D1D2
M1D1
C
D1
M1
M1D2
e
Flavonoids
Coumarins
Lnaps
Ac2PIM2
Flavonoids
V79
Flavonoids
Coumarins glycosides
Glycerophospholipid
Phosphatidic acids
Macrolides
Limonoids
Cinnamic acid
Oligosaccharides
Glycoglycerolipid
DLCL
Fatty acyl
Glycoglycerolipid
Phosphatidic acids
Glycoglycerolipid
V79
Rotenones
Terpene glycosides
M1D12
D2
D1D2
M1D1
C
D1
M1
M1D2
f
Methoxyphenols
N-Fructosyl amino acid
Dihexosylceramide
Phosphatidic Acid
Alkaloids
Tetragydrofurans
Medium-chain fatty acid
N-acyl-alpha amino acid
Anilides
Anilides
Acrylic acids
N-acyl-alpha amino acid
Alpha amino acid
Dilysocardiolipin
Triazinethiones
Pyridinecarboxylic
Benzyl alcohols
Gamma butyrolacton
Medium-chain fatty acid
Dipeptides
Purine nucleosides
Pyridinesulfonamid
Phenylpropanes
Trihexosylceramide
Nitrobenzoic acid
Dicarboxylic acids
Dihexosylceramide
Cinnamic acid
Lysophosphatidylinositol
Phosphatidic Acid
Indolyl carboxylic acid
Cardenolide glycosides
Trihexosylceramide
Benzophenones
M1D12
D2
D1D2
M1D1
C
D1
M1
M1D2

Table S1: This table presents *p*-values comparing metabolome diversity metrics and metabolomic/trait-level responses for soil memory (SM). Metabolome analysis was performed in both positive (P) and negative (N) ionization modes. Significant results ($p < 0.05$) indicate treatment-induced effects and are in bold.

**Treatment**	**Richness (*p*-value)**		**Shannon (*p*-value)**		**Hill Even (*p*-value)**		**Uniqueness (*p*-value)**		**Metabolites-treatment (*p*-value)**		**Traits-treatment (*p*-value)**
	P	N	P	N	P	N	P	N	P	N	
**SM**	0.077	0.118	0.45	0.77	0.63	0.67	0.073	0.038	**0.025**	**0.041**	0.131

Table S2: Table showing effects of M1, D1, D2, i.e., MeJA memory, drought memory, and current drought respectively, and their interactions on Hill Eveness. The variation explained is shown for the significant predictors ($p < 0.05$).

Treatment	Hill Eveness			
	Positive mode		Negative mode	
	P-value	Variation explained	P-value	Variation explained
D1	0.01	10.66	0.97	-
D2	0.357	-	0.01	14.35
M1	0.007	19.78	0.37	-
D1×D2	0.8	-	0.43	-
D1×M1	0.011	10.47	0.36	-
D2×M1	0.489	-	0.89	-
D1×D2×M1	0.167	-	0.897	-
